# A Comparative Study on Volatile Compounds and Sensory Profile of White and Red Wines Elaborated Using Bee Pollen versus Commercial Activators

**DOI:** 10.3390/foods10051082

**Published:** 2021-05-13

**Authors:** Antonio Amores-Arrocha, Pau Sancho-Galán, Ana Jiménez-Cantizano, Víctor Palacios

**Affiliations:** Department of Chemical Engineering and Food Technology, Vegetal Production Area, Faculty of Sciences, Agrifood Campus of International Excellence (ceiA3), University of Cadiz, 11510 Cadiz, Spain; pau.sancho@uca.es (P.S.-G.); ana.jimenezcantizano@uca.es (A.J.-C.); victor.palacios@uca.es (V.P.)

**Keywords:** bee pollen, volatile compounds, fermentative activator, odorant activity value, alcoholic fermentation, white wines, red wines

## Abstract

Lack of nutrients in grape may cause problems for a proper alcoholic fermentation process, resulting in an altered aromatic profile of the wines. To avoid this situation, commercial winemakers often use fermentation activators, which are usually combinations of ammonium salts, inactivated yeast and thiamine. In addition, it has been shown that bee pollen addition to the grape can help to improve fermentation, resulting in better volatile compound profile of wines responsible for sensory quality. For this reason, the aim of this research work was to carry out a comparative study using bee pollen versus commercial fermentation activators in white and red winemaking. The same dose of bee pollen and commercial activators (0.25 g/L) were used in all experiments. Volatile compounds were analyzed by gas chromatography–mass spectrometry, odor activity values were determined to assess odorant impact of various volatile compound families, and finally a descriptive sensory analysis was carried out. Then, the triangular test and the ranking assay were used to identify perceptible differences as well as preference among the wines elaborated. Compared to commercial activators, bee pollen wines increased volatile compound formation, mainly higher alcohols, esters, and terpenes, enhancing fruity and floral odorant series. On the other hand, triangular test showed significant differences between wines, and the ranking assay showed a greater preference for bee pollen wines.

## 1. Introduction

Wine flavor is a combined perception of visual attributes, taste, and aroma, with the aroma being the most responsible for the global perception of wines [1]. The role of wine aroma can be a determining factor in consumer’s preferences [2,3,4] and is one of the key aspects to be taken into account during winemaking. Wine aroma compounds can be grouped according to their origin: varietal aromas are found in grapes, fermentative aromas are derived from the alcoholic and malolactic fermentation, and ageing aromas are obtained during ageing or storage [5]. However, many of the volatile compounds generated during alcoholic fermentation (especially esters, higher alcohols, volatile acids, and various terpenoids and thiols), produced via the metabolic activity of *Saccharomyces cerevisiae*, account quantitatively for biggest fraction of the total aroma composition of wine [1,5,6].

Fermentative yeasts require adequate nutrient levels in the must for proper alcoholic fermentation. A lack of nutrients in grape musts could induce a rapid growth of non-target microorganisms and a displacement of the starter yeast strains could result in sluggish or stuck fermentations [7,8]. Nitrogen or certain vitamin (thiamine and pantothenic acid) deficiencies in grape must could induce problems during the alcoholic fermentation process, resulting in significant sensory defects in final wines [9,10,11,12,13].

In the current wine industry, the use of dehydrated yeast cultures (commercial active dry yeast, ADY) is an extended practice for winemaking [14]. Commercial yeast strains are usually implicitly linked to a high nutrient demand for proper inoculum implantation and subsequent alcoholic fermentation [15]. For a correct development of alcoholic fermentation, these yeasts need to be supplemented with mix of macronutrients, such as sugars, free amino nitrogen, phosphorus, potassium, and magnesium, and micronutrients, such as calcium, copper, iron, manganese, and zinc [14]. In this regard, commercial yeast activators are used in wineries to correct nutritional deficiencies in grape musts [16,17,18,19,20] in order to supply yeast nutritional necessities and to avoid the appearance of problems during alcoholic fermentation [21,22,23].

Bee pollen is a natural source of proteins, essential amino acids, lipids, fatty acids, sterols, phospholipids, carbohydrates, carotenoids, and polyphenols [24,25,26,27,28,29,30]. Previous published research works proposes the use of bee pollen as an alternative to the use of commercial fermentation activators. The effects of its use at different doses have been studied on the sensory profile of white [31] and red [32] wines, showing that bee pollen improves the sensory profile of wines when used at low doses. For this reason, this research study proposes a comparative study between bee pollen use versus commercial activators employed for white and red winemaking.

## 2. Materials and Methods

### 2.1. Experimental Procedure

The grape must of the white variety Palomino Fino was obtained from the Cooperativa Andaluza winery, Unión de Viticultores Chiclaneros of Chiclana de la Frontera (36.426067, −6.148189, 50 m above sea level) and the Riesling grape must from a private winery of Jerez de la Frontera (36.666594, −6.114847, 50 m above sea level). The red grape variety, Tintilla de Rota, was harvested from the private winery Luis Pérez in Jerez de la Frontera (36.700167, −6.192778, 100 m above sea level). All the vineyards were located in southern Andalusia, Cádiz (Spain), and were grown under warm climate conditions over an albariza (limestone) soil.

Once the white grape musts were obtained, they were added with 90 mg/L of potassium metabisulphite and racked for 24 h at controlled temperature (10 °C). Red grapes were destemmed and crushed, and the resulting paste was dosed with 25 mg/L K_2_O_5_S_2_ (Sigma-Aldrich Chemical S.A., Madrid, Spain). The white grape must and the paste (pulp + skins) of Tintilla de Rota were placed in 5-liter glass fermenters jacketed for temperature control. For each vinification (bee pollen or commercial activator addition), 9 simultaneous fermenters were prepared including control. Each experiment was conducted in triplicate (*n* = 3).

Bee pollen has been widely employed by the research group in several studies, showing good results in both white [31,33,34] and red [32,35] vinification. For this reason, the dose of this comparative study was estimated as 0.25 g/L of bee pollen, previously crushed and preserved under dark and desiccation conditions. For white vinifications, a commercial activator SUPERSTART^®^ BLANC (Laffort, Bordeaux, France) was used, with a specific formulation for white wine vinification conditions. For the red wine, SUPERSTART^®^ ROUGE (Laffort, Bordeaux, France) was used, a preparation based on inactivated yeasts and autolysates of selected yeasts that is rich in vitamins, minerals, fatty acids, and sterols (er-gosterol) and adapted in particular for red wine production. In both cases (white and red), vinifications with commercial activators were carried out at a dose of 0.25 g/L, equal to the dose of bee pollen used.

Alcoholic fermentation (AF) was carried out using a commercial yeast starter *Saccharomyces cerevisiae* Lalvin 71B^®^ (Lallemand, Barcelona, Spain) under controlled temperature conditions (20 °C). In the red wine vinification, once AF was finished, malolactic fermentation (MLF) was carried out using a commercial strain of a lactic acid bacteria (LAB) *Oenococcus oeni* S11B P2 Instant (Laffort, Bordeaux, France) at the recommended dosage (1 g/hL).

### 2.2. Aroma Compounds and Odorant Activity Values (OAV)

Major volatile compounds were determined by gas chromatography with flame ionization detection (GC-FID) using 4-methyl-2-pentanol as internal standard and standard calibration to determinate retention times and calibration curves. On the other hand, the minority volatiles were identified and quantified by semiquantitative analysis, using 1-heptanol (Sigma-Aldrich Química, S.A., Madrid, Spain) as internal standard, assuming a response factor equal to one according to the methodology described by Amores-Arrocha et al. [31]. To analyze the odorant activity value (OAV) on final wines, we calculated the ratio of the concentration of each compound and its perception threshold. The same methodology described by Amores-Arrocha et al. [31,32] and aroma descriptors previously published by several authors were employed for this purpose [16,18,20].

### 2.3. Sensory Testing

All tasting sessions were conducted in a temperature-controlled tasting room at the University Institute of Viticulture and AgriFood Research (IVAGRO, Puerto Real, Cádiz). Each taster was located in an individual booth with controlled lighting that was separated from the rest of the judges by panels to avoid possible interactions. Each judge was provided with the same amount of wine in standard ISO 3591 (1997) [36] glass cups covered with a glass lid, and temperature in the room was controlled in order to avoid the evaporation of volatile compounds (20 ± 2 °C). Each tasting session (descriptive analysis, sorting test, and triangular test) took place on different days.

#### 2.3.1. Sensory Descriptive Analysis

A total of 20 judges, previously trained and experienced in sensory evaluations of white and red wines, were involved in the descriptive sensory analysis. All wine samples were randomly coded with three digits and presented disorderly. Each judge was assigned a tasting sheet to evaluate the intensity of different attributes on a scoring scale of 0–10 points. Attributes evaluated during the tasting sessions were previously selected, taking into account the tasting descriptors for white and red wines according to Jackson (2009) [37].

#### 2.3.2. Classification Test

The ranking test is a sensory evaluation technique whereby a series of samples can be ordered according to established criteria. For this test, judges were instructed to order the wines samples by hedonic preference in order to determine the existence of differences between them. Once samples were classified, an increasing score was assigned according to the order of preference and the sums of the rankings of the samples were calculated for each judge, rejecting the null hypothesis (H_0_) when there was no difference between the samples. Page’s test value was applied for a comparison with respect to a known order. Observing the results pre-established by ISO 8587:2006 AENOR (2010) [38], we find that H_0_ will be rejected if F test > F, considering the number of judges, the number of samples, and the risk assumed; therefore, it may be concluded if there are consistent differences between the ranking of the samples. A total of 29 judges were used to carry out the ranking test.

#### 2.3.3. Triangular Test

To determine any perceptible variances between wines elaborated (control, pollen and commercial), we applied the ISO 4120:2007 AENOR (2008) [39] triangular test. This test allows us to know if there is any perceptible difference between three samples, regardless of the possible nature of these differences [40]. In the triangular test, triad samples are presented simultaneously, where two of them are always identical and one is different. Panelists must indicate in each case which sample is the different one. In this sense, it is hypothesized that the probability of choosing the different sample when there is no difference between them is 1/3 (H0: Pt = 1/3). In this modality, samples were presented to the judges in different position sequences (AAB, AAC, BCC, ABB, ACC, and BCC). After a judges’ evaluation, the next round of samples was placed in turn in the order of the sequence, and therefore during the evaluation sessions, all samples were presented in equal number and randomly. According to the standard, if the number of correct answers is greater than or equal to the number established in the table (corresponding to the number of judges and the risk level in the test), it can be concluded that there are perceptible differences between samples. The triangular test was carried out with the participation of 28, 27, and 26 judges for Palomino Fino, Riesling, and Tintilla de Rota wines, respectively.

### 2.4. Data Analysis

Means and standard deviations were determined by two-way ANOVA analysis and Bonferroni’s multiple range test (BSD), considering *p* < 0.05 as significant (GraphPad Prism version 6.01 for Windows, GraphPad Software, San Diego, CA, USA). All tests were performed in triplicate (*n* = 3). For principal component analysis (PCA), SPSS 24.0 statistical software package (SPSS Inc., Chicago, IL, USA) was employed.

## 3. Results and Discussion

### 3.1. Influence of Bee Pollen and Commercial Activators on Volatile Compounds

Table 1, Table 2 and Table 3 shows in comparative terms the effect of the addition of bee pollen, commercial activator, and control on the profile of the volatile compound families of Palomino Fino, Riesling, and Tintilla de Rota wines, respectively. It can be observed that bee pollen use significantly increased the total content of volatile compounds, resulting in an increment over the control by 3, 4, and 12% in the Palomino Fino (Table 1), Riesling (Table 2) and Tintilla de Rota (Table 3) wines, respectively (*p* < 0.05, ANOVA). However, a marked decrease in the production of volatile compounds in Palomino Fino and Riesling wines, around 11 and 22%, respectively, was observed with the commercial activator. In Tintilla de Rota wines, the values remained at the same levels as control (Table 3).

As can be seen, the higher alcohol levels in both white and red wines reached higher values using bee pollen, in comparison with the control and the synthetic activator, while the volatile acid content decreased in all wines with pollen, possibly due to the richness of bee pollen in fatty acids [15]. This decrease in volatile acids with pollen use favors the aromatic profile of the wines, since most of the compounds of this family have aromas within the fatty and rancid series, which can negatively affect the aromatic quality of the wines. Regarding C6 alcohols, the results showed an increase in Palomino Fino wines with pollen, in comparison with the control. In Riesling and Tintilla de Rota wines, significant reductions were observed with the use of pollen and commercial activators. In addition, alcohols decreased in all pollen and commercial activator wines compared to their controls. Considering terpenes and esters, all the bee pollen wines experienced a significant increase in their content, reaching much higher values in the case of esters. With respect to aldehydes, an increase in this family of compounds was only observed in Riesling white wines, noting the very low levels reached for both varieties. Lastly, phenols, only detected in red wines, showed an opposite behavior between pollen and synthetic activator. In red pollen wines, over 26% increase was observed in comparison with the values obtained in controls and wines with commercial activator.

#### 3.1.1. Major Alcohols and Methanol

The majority alcohol levels in wines (white and red) achieved greater values with the use of bee pollen compared to controls and commercial activators (Table 1, Table 2 and Table 3), especially Tintilla de Rota red wines (Table 3). In red wines, higher alcohols were significantly raised in similar proportions as in previous study [32], except for 2-methyl-1-propanol, which had the lowest concentration (Table 3). This behavior was also observed in white wines, mainly due to the increase of 3-methyl-1-butanol in Palomino Fino and 2-propanol and 3-methyl-1-butanol in Riesling wines [31]. Regarding methanol, we observed a slight increase in its concentration (0.15 %) in Palomino Fino pollen wines compared to the control, while the commercial activator wines decreased in methanol production (Table 1). The same behavior was observed in a previous research on different bee pollen doses in Palomino Fino wines [31]. On the other hand, in Riesling (Table 2) and Tintilla de Rota wines (Table 3), we observed a considerable decrease in methanol concentration, both with pollen and with the commercial activator, being more marked in red wines.

#### 3.1.2. Volatile Acids

The volatile acid content was lower in all bee pollen wines (Table 1, Table 2 and Table 3) as a consequence of pollen fatty acid richness [15]. However, the behavior of wines with commercial activator differs for the white wines. In Palomino Fino wines, a decrease was observed (Table 1), while in Riesling wines (Table 2), there was a significant increase. In the case of Tintilla (Table 3), the reduction in acid content was mostly related to hexanoic, octanoic, and decanoic acids. The volatile acid family compounds decreased as a result of pollen use. This family of compounds disfavors the aromatic profile of the wines, as the majority volatile acids present aromas within the fatty and rancid series by altering the aromatic quality of wines [31,32].

#### 3.1.3. C-6 Alcohols

In Palomino Fino wines (Table 1), the production of C6-alcohols decreased compared to the control by 47.08% with the use of pollen and 25.85% with the commercial activator, mainly due to 1-hexanol. On the contrary, in Riesling (Table 2), this family of compounds increased by 47.30% and 21.07% with pollen and commercial activator, respectively. In Tintilla de Rota wines (Table 3), the C6-alcohols levels decreased significantly using pollen, although with the commercial activator, there was a greater decrease compared to the control.

#### 3.1.4. Alcohols and Terpenes

Alcohols decreased significantly compared to the control, both in pollen-elaborated wines and with commercial activator, highlighting the decrease in the commercial activator wines. The main responsible compounds for the decrease in this family in wines with pollen were 1-pentanol and 1H-indole-3-ethanol for all wines (Table 1, Table 2 and Table 3). In terms of the commercial activator, the main responsible compounds were 1-pentanol in Palomino Fino and 2-phenylethanol in Riesling wines. For Tintilla de Rota wines, the decrease in alcohols was caused by 1-pentanol and 1H-indole-3-ethanol. Regarding the terpene content, it was clearly observed that terpene levels were significantly higher in all pollen-elaborated wines, supporting pollen’s ability to transfer this family of compounds to the wine.

#### 3.1.5. Esters

As for esters, all wines showed a significant increase in their ester content, reaching a 10-fold increase over control wines. In Palomino Fino (Table 1) and Riesling (Table 2) wines, bee pollen produced more esters than the commercial activator, while for Tintilla de Rota wines, the values were similar to each other (Table 3). These results could demonstrate that pollen contributes significantly to the synthesis and formation of esters, intensifying fruity and floral aromatic series in wines.

#### 3.1.6. Aldehydes

The levels of aldehydes reached for the white wines of both varieties were very low. Riesling wines showed an increase (Table 2), while Palomino Fino wines decreased with both bee pollen and commercial activator (Table 1). The same behavior was observed for Tintilla de Rota (Table 3) as for Palomino Fino white wines. Moreover, aldehyde levels were significantly lower in all pollen-elaborated and commercial activator-elaborated wines compared to the control, caused in particular by pronounced reductions in acetaldehyde. However, the levels of benzene-acetaldehyde, nonanal, and 3-methylbutanal were higher compared to the control wines.

#### 3.1.7. Phenols and Minority Compounds

The phenol content, only detected in Tintilla de Rota wines, showed an opposite behavior between bee pollen and commercial activator wines. Pollen contributed more than 26% to the phenol levels of the control and the commercial activator wines. These compounds could possibly play a role in the spicy character of wines [41].

Among the families of minority compounds also detected in Tintilla de Rota, represented by thiols, acetals, and norisoprenoids, we observed that bee pollen and commercial activator addition enhanced thiol and norisoprenoid formation.

### 3.2. Principal Component Analysis of Volatile Compounds (PCA)

**Figure 1:**
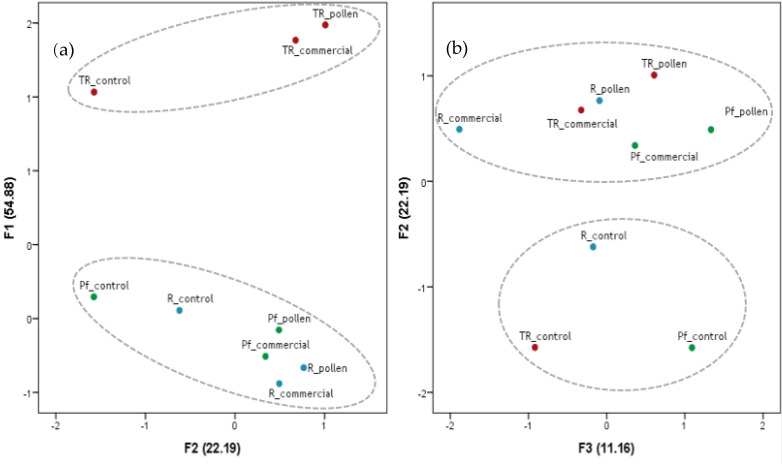
Principal component analysis of Palomino Fino (Pf, green), Riesling (R, blue), and Tintilla de Rota (TR, red) wines fermented with bee pollen against a commercial activator and control.

Table 4 shows the loadings for the principal component analysis (PCA) factors extracted on the dataset corresponding to the analyzed families of volatile compounds in the white and red wines: higher alcohols, methanol, acids, C6 alcohols, alcohols, phenols, terpenoids, esters, aldehydes, acetals, norisoprenoids, and lactones. Three factors were extracted from the data analysis, which explained over 88% variance data. Factor F1 was in positive correlation with most volatile minority compounds (thiols, methanol, acetals, norisoprenoids, lactones, and phenols) characteristic of Tintilla de Rota wines and negatively with C6 alcohols. Terpenes were also positively included in F1, with lower loadings. This effect was probably related to the influence of the grape skins on this group of compounds during Tintilla de Rota vinification. As can be seen in Figure 1a, all white wines showed negative F1 values, with the lowest values for the commercial activator and pollen wines. On the contrary, all red wines showed positive values (>1), highlighting bee pollen wines with the highest levels, supporting the enhancing influence on the minority compounds involved in Tintilla de Rota’s varietal expression.

Factor 2 (F2) was positively correlated with the volatile compounds mostly responsible of the aromatic profile of wines (esters and terpenes) and negatively with aldehydes and alcohols. F2 therefore grouped the family of aromatic compounds most influenced by the presence of bee pollen. According to the results obtained, the use of bee pollen mainly promoted ester production and the transfer of terpenes, and decreased alcohol and aldehyde formation. Bee pollen was highly correlated with an increase in compounds responsible of aromatic notes of fruits and flowers, qualities determining during the sensory analysis of the wines. Another effect observed in F2 was a correlation with terpenes and derivatives, mainly as result of the synergistic effect of pollen and skins (red wines) upon this volatile compound family. Figure 1a,b shows the aroma-enhancing effect of pollen during vinification in white and red wine.

All wines with bee pollen and commercial activator showed positive values in F2, highlighting pollen-elaborated wines. This would indicate that the use of bee pollen in winemaking enhances, on one hand, the formation and, on the other hand, the extraction of compounds contributing to the aromas in wines.

The third factor (F3) mainly correlated and grouped higher alcohols, the majority volatiles in the wines. Pollen had a clear effect on the behavior of this compound family with respect to both wines, white and red, enhancing higher alcohol production. Figure 1b shows the two aromatic factors (F2 and F3) where bee pollen exerted a certain effect. As can be seen, all the wines with bee pollen had the highest F3 levels in their respective series compared to the control and to the commercial activator wines.

### 3.3. Description of Odor Activity Value (OAV)

Table 5 and Table 6 show the values of the sum of the OAV of each aromatic series, as well as the sum of all the series (ΣOAV_T_).

The sum of the OAV groups together the different aromatic series in which the different volatile compounds are associated with their perception threshold according to the literature. In general, all the wines with pollen showed the highest ΣOAV_T_ values, with markedly higher variances in pollen wines versus the control and commercial activator. This can be explained by the fact that the wines with pollen had the highest levels in floral and fruity odor series among all the wines studied.

The commercial activator also enhanced or intensified the fruity and floral series for Riesling and Tintilla de Rota wines, although to a minor extent compared to bee pollen. The exception was Palomino Fino wines, where the fruity and floral odorant series values in the wines with commercial activator were very similar to the control.

**Figure 2:**
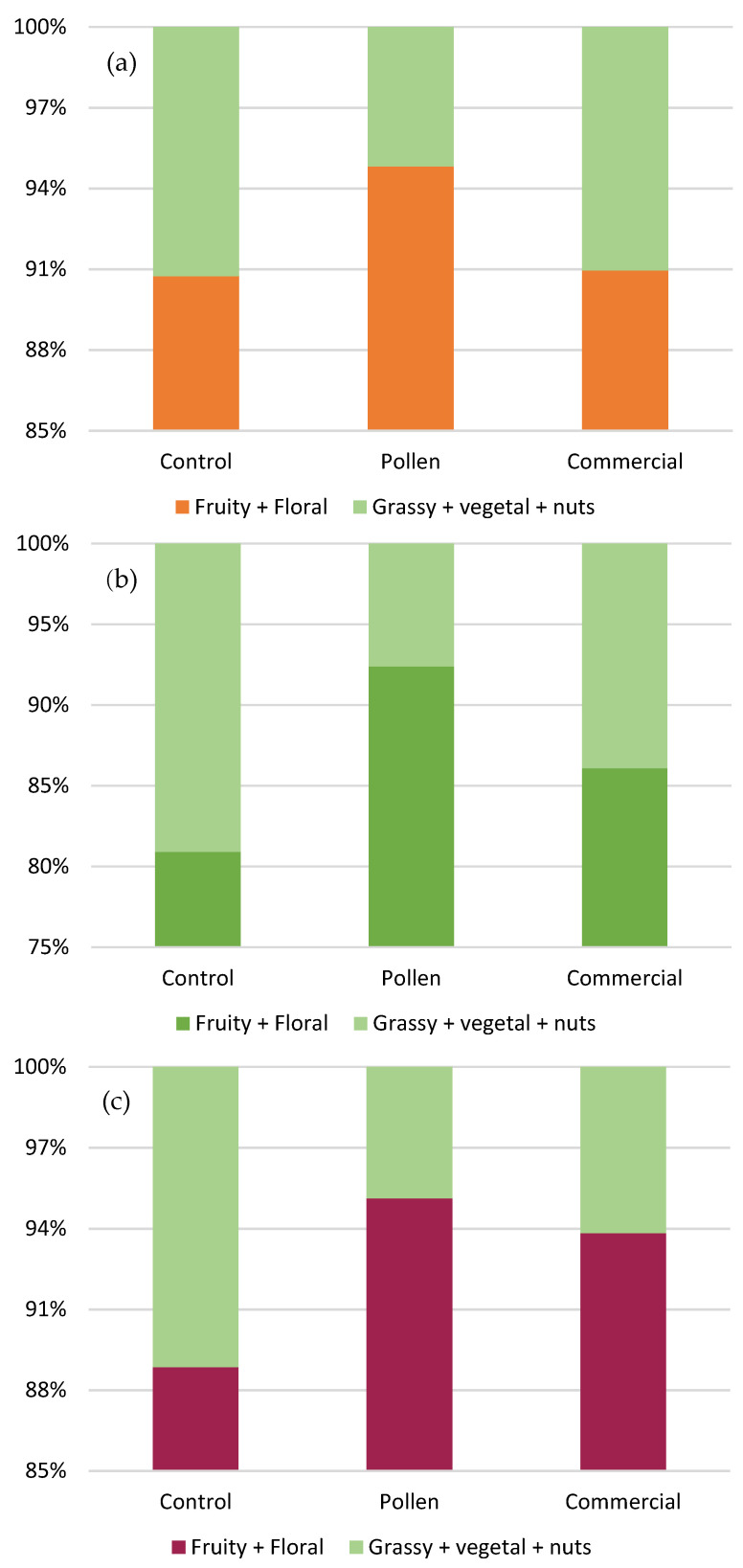
Participation percentage of the odorant series (fruity, floral, fatty, herbaceous, and dry fruit) in the white wines of Palomino Fino (**a**) and Riesling (**b**), and in Tintilla de Rota red wines (**c**).

Figure 2 show the increment percentage of “positive” odorant series (fruity + floral) versus “negative” odorant series (fatty + herbaceous + raisin fruit (nuts)) for Palomino Fino (Figure 2a) and Riesling (Figure 2b) white wines and Tintilla de Rota red wines (Figure 2c).

The greatest contribution to the aromatic profile in all cases was made by the “positive” odorant series, whose values were above 93–95% in the wines with bee pollen, followed by the commercial activator, with values above 83–93%, and finally the control with 81–90%.

Therefore, from a general point of view, it could be pointed out that pollen managed to enhance the formation of volatile compounds with positive odoring effects (fruit and flowers), as opposed to volatile compounds with negative odoring effects (fatty).

### 3.4. Descriptive Sensory Analysis

A sensorial analysis of all the wines was carried out to describe the organoleptic attributes that described them in detail. A specific tasting sheet was used for the white wines and another sheet for the red wines. In order to carry out the comparative study in detail, we made a distinction between the general tasting attributes and the specific attributes for each type of wine.

#### 3.4.1. General Attributes in White Wines

**Figure 3:**
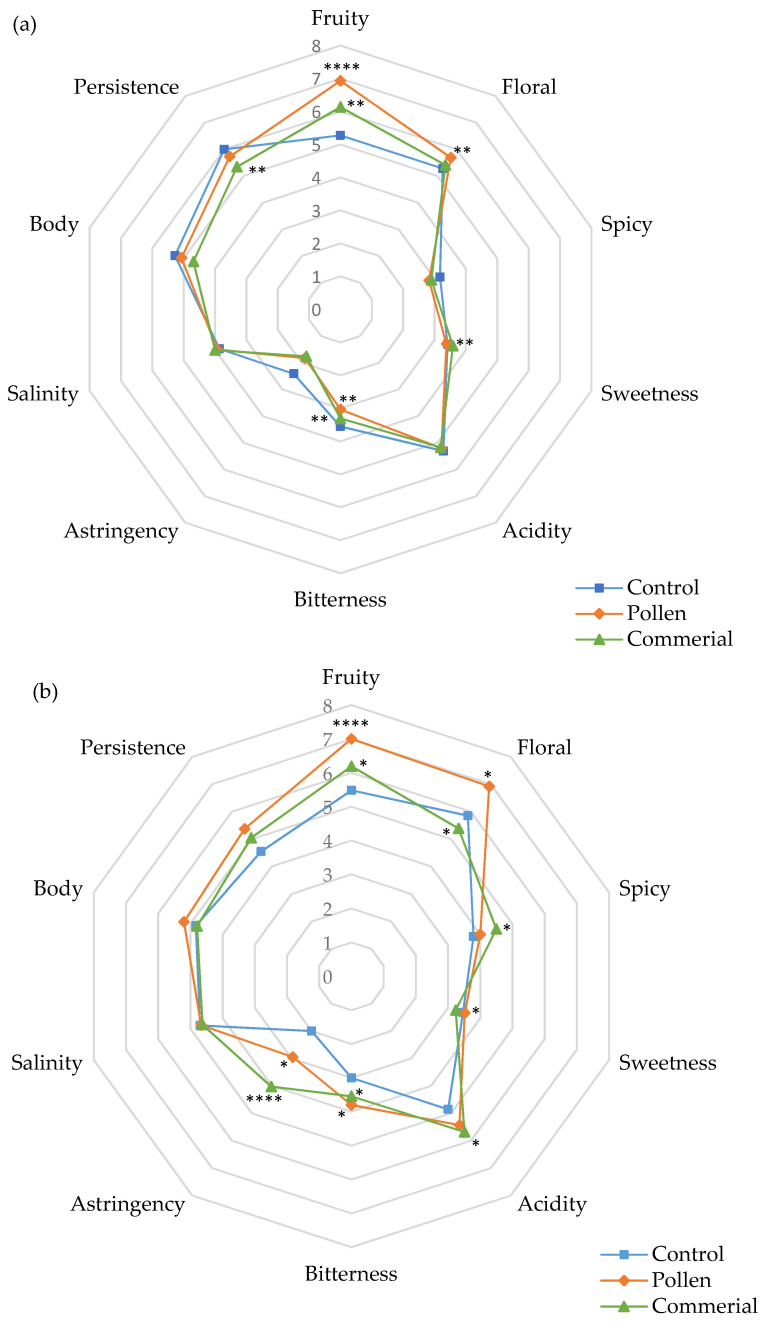
General attributes of the sensory analysis of Palomino Fino (**a**) and Riesling (**b**) wines (control, pollen, and commercial activator). * Statistical significance for two-way ANOVA (BSD test) (* *p* < 0.05, ** *p* < 0.01,, and **** *p* < 0.0001).

**Figure 4:**
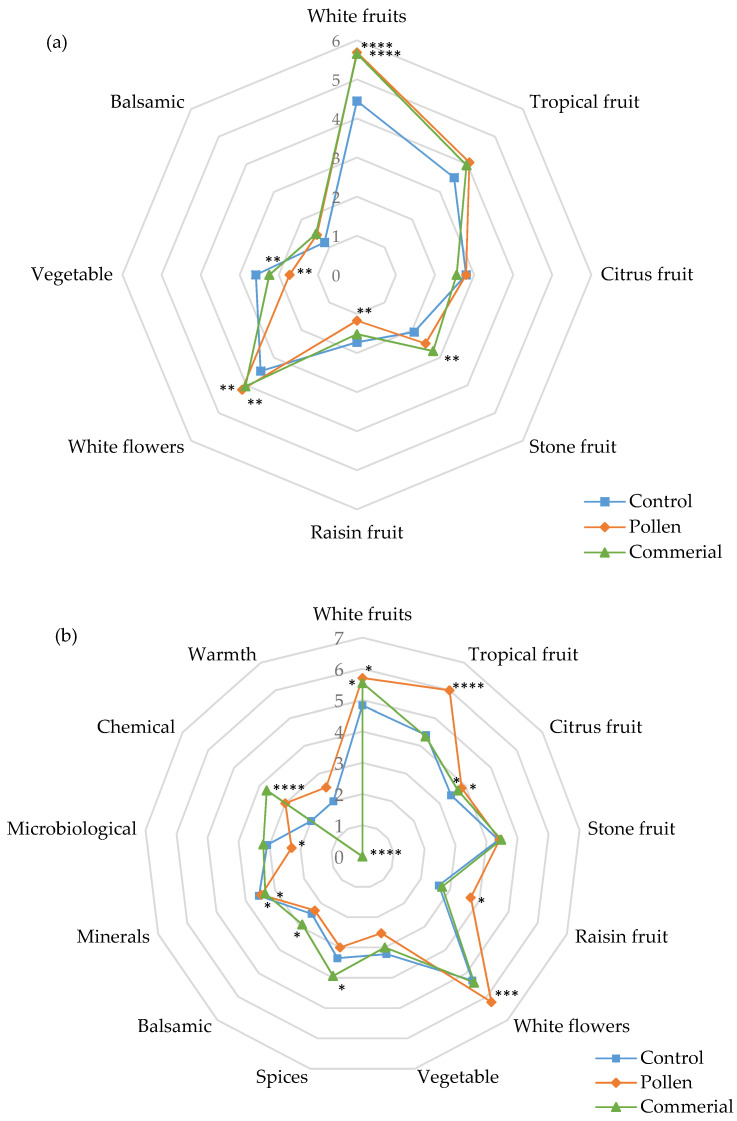
Specific attributes of sensory analysis of Palomino Fino (**a**) and Riesling (**b**) wines (control, pollen, and commercial activator). * Statistical significance for two-way ANOVA (BSD test) (* *p* < 0.05, ** *p* < 0.01, *** *p* < 0.001, and **** *p* < 0.0001).

Figure 3 and Figure 4 show the cobweb diagrams corresponding to the sensory profile of the general attributes for Palomino Fino and Riesling white wines, correspondingly (fruity, floral, and spicy character on the nose; sweetness, acidity, bitterness, astringency, salinity, body, and persistence). As can be seen, in both cases, wines with pollen scored significantly higher in the fruit and floral attributes related to the control and the commercial activator, whereby control wines obtained the lowest scores. These scores verified the OAV results and probably reflect that pollen specifically enhances the synthesis of esters during alcoholic fermentation.

In Palomino wines, floral character scored no significant differences between control and commercial activator wines (Figure 3a). However, control wines showed the best scores compared to the commercial activator in Riesling wines (Figure 3b).

Regarding the sweet mouthfeel attribute, the control and pollen wines showed lower scores compared to the wines with commercial activator, while in Riesling, control and pollen wines scored slightly higher compared to the wines with commercial activator. In terms of acidity, all the samples showed moderate acidity levels, and only the commercial activator and pollen wines were slightly higher than the control. No wine stood out for its bitter character; however, the control in Palomino Fino scored significantly higher than pollen and commercial activator, while in Riesling, the behavior was the opposite. Astringency of these wines was rated very low in general, with only Riesling wines with commercial activator receiving the highest scores. As for the saline character, all the wines showed low values with no significant differences.

Concerning the texture sensation in the mouth, we should note that the wines with the most body were control and pollen in Palomino Fino, and pollen wines in Riesling. Synthetic activator wines obtained the lowest scores in both cases. Moreover, it was the same samples that presented significantly higher persistency in Palomino wines, while pollen and commercial activator Riesling wines were better scored.

#### 3.4.2. Specific Attributes in White Wines

The specific attributes (white fruit, tropical fruit, citrus fruit, stone fruit, dried fruit, flowers, spicy aromas, balsamic, etc.) evaluated in the sensory analysis are shown in Figure 4. A detailed analysis of specific attributes of the olfactory sensory phase among white wines showed that, in general, Riesling (Figure 4a) presented a more complex and richer sensory profile than Palomino Fino wines (Figure 4b).

While in Palomino Fino wines, white fruit notes (apple and pear) were significantly intensified, in Riesling, in addition to white fruit, tropical fruit (banana, melon, pineapple, passion fruit) and citrus notes (tangerine and lime) were increased compared to the commercial activator and the control. Palomino Fino wines with commercial activator and pollen showed higher intensity in stone fruit and lower intensity in raisin fruit compared to the control, despite the latter attribute scoring very low in all cases. Only a slight increase in raisin fruit notes was observed in the Riesling wines with pollen.

In Palomino Fino pollen and commercial activator wines, the highest scores were attributed to the intensity of white flowers (orange blossom, jasmine), while the rest of the attributes were assessed by tasters with very low scores. The intensity of white flowers was higher in Riesling wines than in Palomino Fino, in line with the increases observed in this odor series in the OAV analysis. Riesling wines made with the commercial activator showed increases in balsamic, microbiological, and chemical notes, not observed in pollen wines. This behavior suggests although a more complex sensory profile was achieved with the Riesling variety, the intensity of these minority attributes seemed to be integrated in a well-balanced way. Regarding the warmth attribute, Riesling wines with pollen had a warmer mouthfeel, possibly related to the slight increase in body and persistence noted.

#### 3.4.3. General and Specific Attributes in Red Wines

**Figure 5:**
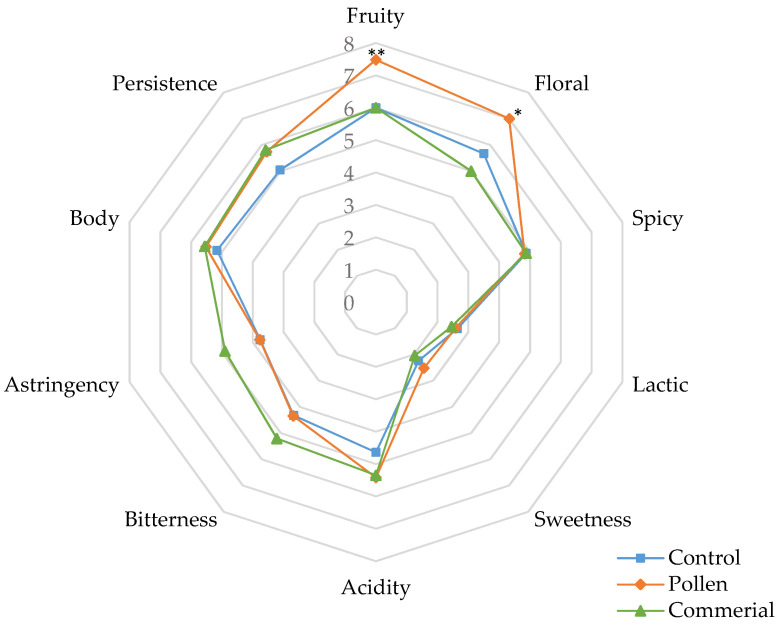
General attributes of the sensory analysis of Tintilla de Rota wines (control, pollen, and commercial). * Indicates level of significance for two-way ANOVA (BSD test) (* *p* < 0.05 and ** *p* < 0.01).

Figure 5 represents the results obtained for the general attributes after the sensory analysis of the Tintilla de Rota red wines (control, pollen, and commercial).

In general, Tintilla de Rota wines fermented with bee pollen presented significantly higher scores in fruity and floral attributes in comparison to the control and the commercial activator. No variation in sweetness intensity was observed in any of the cases. However, a higher score was observed in the acidity intensity in wines with bee pollen and commercial activator. Bitterness and astringency sensations together showed very different behavior in the wines. The wines with pollen and control were the ones with the lowest scores in both attributes. As with the Riesling wines, red wines with pollen and commercial activator were the most intense in the attributes of mouthfeel, body, and persistence.

**Figure 6:**
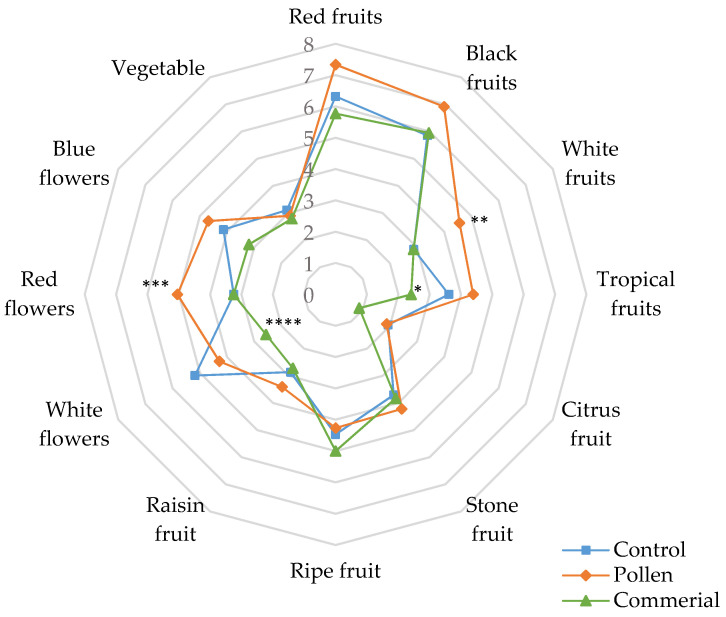
Specific attributes of the sensory analysis of Tintilla de Rota red wines (control, pollen, and commercial). * Statistical significance for two-way ANOVA (BSD test) (* *p* < 0.05,** *p* < 0.01, *** *p* < 0.001 and **** *p* < 0.0001).

Specific attributes evaluated in the sensory analysis of the Tintilla de Rota red wines (control, pollen, and commercial) are shown in Figure 6.

Considering the specific olfactory attributes, all red wines with bee pollen showed significantly more richness and diversity in the fruity profile (blackberries, raspberries, cherries, apple, as well as melon, mango, passion fruit, tangerine, and lime notes) (ANOVA *p* < 0.05) compared to control and commercial activator wines. No substantial differences were observed in stone fruit, ripe fruit, and raisin fruit aromas. In relation to the floral attributes, the wines with pollen scored best in their notes of blue flowers (violets and lilacs) and red flowers (roses), and to a lesser extent in white flowers, where the control stood out. Both types of wines were better scored than the commercial activator. All other attributes showed no significant differences between the wines.

### 3.5. Classification and Triangular Test

Once characterized by attributes (olfactory, taste, and texture), wines were tested by the classification test (ISO 8587:2006 AENOR, 2010) [38] and the triangular test (ISO 4120:2007 AENOR, 2008) [39].

Classification test results for white and red wines elaborated with pollen versus a commercial activator and control are shown in Table 7. Triangular test results for the obtained values of alpha risk (α), number of tasters, and percentage of tasters who identified the different wines in each category are shown in Table 8.

According to results reported in Table 7 and Table 8, we can conclude that there were consistent differences between the classification of wines:

- In Palomino Fino wines, according to classification test, the pollen and control wines were significantly preferred against the commercial activator. On the basis of a triangular test, we found that commercial activator wines were significantly different from the control (α = 0.2) and the pollen wines (α = 0.01). No significant differences were found between the control and pollen.
- In Riesling wines, pollen wines were significantly preferred over all others, distantly followed by the commercial activator and lastly by the control wines. According to the triangular test, significant differences were obtained in all comparisons.
- In Tintilla de Rota wines, regarding the classification test, similar results were obtained to Riesling, with a significant preference for pollen wines, followed by commercial activator and, lastly, control wines. On the basis of the results obtained from the triangular test, as in Riesling, we found that all the wine samples showed differences among themselves.

The largest percentage of tasters were able to identify the differences between the white wines. In Palomino Fino wines, this was by comparing pollen against the commercial activator, and in Riesling, between control and commercial.

According to the triangular test results for Tintilla de Rota red wines, we found that there was similar behavior of all the wines in general. The control–pollen confrontation obtained a slightly higher percentage than the rest.

## 4. Conclusions

Bee pollen use at a dose of 0.25 g/L versus a commercial activator led to an improvement in the formation of volatile compounds, especially higher alcohols, esters, and terpenes, resulting in an OAV increase of fruit and floral odor series.

Analyzing the classification and triangular test results together, along with the detailed information provided by the sensory analysis, we concluded that:

- The high percentages of tasters able to identify the different wines during the triangular test sessions proved the existence of significative differences amongst wines elaborated with pollen, commercial activator, and control.
- Results of the classification test showed a significant preference in most cases for the wines made with pollen, both in whites and reds.

Finally, taking into account the descriptive sensory analysis results, we were able to determine that these organoleptic differences between the wines were produced by improvements in the aromatic intensity of the sensory attributes corresponding to fruity and floral aromas. For this reason, the wines vinified with bee pollen were the best rated by the tasters.

Therefore, bee pollen could be considered as a valid and natural alternative to enhancing aromatic profile of fruits and flowers in young white and red wines.

## Figures and Tables

**Table 1:** Volatile compound concentration (µg/L) in Palomino Fino white wines (control, pollen, and commercial activator).

Volatile Compound	Control	Pollen	Commercial
***Higher alcohols***			
2-Propanol	419,790.9 ± 7150.0 ^a^	400,774.7 ± 14,870.0 ^a^	404,816.4 ± 2700.0 ^a^
1-Propanol	7851.7 ± 80.0 ^a^	4734.1 ± 100.0 ^b^	4864.3 ± 60.0 ^b^
2-Methyl-1-propanol	29,588.9 ± 1040.0 ^a^	29,268.0 ± 820.0 ^a^	32,853.1 ± 770.0 ^b^
3-Methyl-1-butanol	268,385.1 ± 7622.0 ^a^	347,966.3 ± 915.2 ^b^	229,924.9 ± 4033.7 ^c^
Total	725,616.6 ± 15,892.0	782,743.0 ± 16,705.2	672,458.7 ± 7563.7
% higher alcohols	86.16%	90.20%	90.22%
***Methanol***	38,822.5 ± 1480.0 ^a^	38,881.3 ± 1690.0 ^a^	38,432.2 ± 720.0 ^a^
Total	38,822.5 ± 1480.0	38,881.3 ± 1690.0	38,432.2 ± 720.0
% methanol	4.61%	4.48%	5.16%
***Acids***			
Butanoic acid	32.8 ± 0.7 ^a^	30.2 ± 0.1 ^a^	31.0 ± 0.3 ^a^
3-Methyl-butanoic acid	200.0 ± 0.3 ^a^	199.8 ± 1.8 ^a^	220.5 ± 1.0 ^b^
Hexanoic acid	1811.4 ± 176.4 ^a^	627.2 ± 3.0 ^b^	964.9 ± 18.3 ^c^
Heptanoic acid	37.6 ± 1.5 ^a^	38.4 ± 2.1 ^a^	75.2 ± 3.4 ^b^
2-Hexenoic acid	37.2 ± 0.4 ^a^	42.0 ± 0.8 ^b^	45.2 ± 0.6 ^b^
Octanoic acid	2790.4 ± 131.3 ^a^	1427.5 ± 4.3 ^b^	1899.9 ± 33.6 ^c^
Nonanoic acid	15.3 ± 1.2 ^a^	6.3 ± 0.3 ^b^	5.0 ± 0.1 ^b^
n-Decanoic acid	794.9 ± 14.0 ^a^	638.0 ± 35.8 ^b^	926.1 ± 7.5 ^c^
9-Decenoic acid	248.6 ± 32.3 ^a^	108.1 ± 1.4 ^b^	164.1 ± 8.9 ^c^
Benzoic acid	94.1 ± 1.0 ^a^	80.6 ± 4.7 ^b^	101.2 ± 12.6 ^a^
Phenylacetic acid	9.8 ± 0.3 ^a^	15.0 ± 0.7 ^b^	8.4 ± 0.6 ^c^
Total	6072.0 ± 359.4	3213.1 ± 54.9	4441.5 ± 86.8
% acids	0.72%	0.37%	0.60%
***C-6 Alcohols***			
1-Hexanol	752.0 ± 7.8 ^a^	590.2 ± 4.3 ^a^	613.3 ± 9.0 ^a^
(E)-3-hexen-1-ol	15.2 ± 0.3 ^a^	21.1 ± 1.5 ^a^	16.2 ± 1.0 ^a^
(Z)-3-hexen-1-ol	56.2 ± 64.2 ^a^	105.4 ± 1.7 ^b^	119.4 ± 3.6 ^b^
Total	823.5 ± 72.3	716.6 ± 7.4	748.9 ± 13.5
% C-6 alcohols	0.10%	0.08%	0.10%
***Alcohols***			
3-Penten-2-ol	10.0 ± 0.5 ^a^	18.1 ± 0.2 ^b^	15.2 ± 2.0 ^c^
1-Pentanol	2185.7 ± 7.0 ^a^	1474.5 ± 102.0 ^b^	1170.5 ± 12.4 ^c^
3-Ethyl-2-pentanol	8.2 ± 0.1 ^a^	10.2 ± 0.2 ^b^	7.2 ± 0.2 ^c^
4-Methyl-1-pentanol	10.9 ± 0.6 ^a^	15.7 ± 0.1 ^b^	16.7 ± 0.1 ^b^
3-Methyl-1-pentanol	92.8 ± 5.6 ^a^	114.7 ± 0.3 ^b^	117.6 ± 5.6 ^b^
3-Ethoxy-1-propanol	87.7 ± 0.7 ^a^	79.5 ± 0.7 ^a,b^	76.8 ± 0.4 ^b^
1-Octanol	17.1 ± 1.9 ^a^	43.2 ± 1.0 ^b^	43.0 ± 2.5 ^b^
1-Nonanol	8.9 ± 0.1 ^a^	11.2 ± 1.5 ^b^	12.0 ± 0.7 ^b^
Benzyl alcohol	44.8 ± 1.2 ^a^	78.9 ± 1.6 ^b^	41.6 ± 1.0 ^a^
2-Phenylethanol	1236.5 ± 316.7 ^a^	2119.0 ± 111.5 ^b^	2102.9 ± 6.0 ^b^
1H-Indole-3-ethanol	3240.6 ± 113.5 ^a^	132.9 ± 0.1 ^b^	105.0 ± 3.9 ^b^
Total	6943.3 ± 447.9	4098.0 ± 219.2	3708.5 ± 34.9
% alcohols	0.82%	0.47%	0.50%
***Terpenes***			
Linalool oxide	7.0 ± 0.2 ^a^	10.9 ± 0.9 ^b^	10.1 ± 0.1 ^b^
Linalool	8.1 ± 0.2 ^a^	15.1 ± 0.9 ^b^	10.8 ± 0.5 ^c^
α-Terpineol	11.0 ± 0.3 ^a^	15.8 ± 0.5 ^b^	14.0 ± 0.7 ^c^
β-Citronellol	9.4 ± 0.7 ^a^	13.8 ± 0.8 ^b^	10.1 ± 0.1 ^a^
2,6-Dimethyl-3,7-octadiene-2,6-diol	29.3 ± 2.5 ^a^	32.3 ± 1.5 ^a^	22.3 ± 1.3 ^b^
8-Hydroxilinalool	40.9 ± 0.1 ^a^	48.9 ± 4.2 ^b^	39.4 ± 3.0 ^a^
Total	105.6 ± 3.9	136.8 ± 8.8	106.6 ± 5.7
% terpenes	0.013%	0.016%	0.014%
***Esters***			
Ethyl acetate	11,164.9 ± 90.0 ^a^	11,469.9 ± 20.0 ^a^	8783.8 ± 350.0 ^b^
Ethyl butyrate	5.8 ± 0.7 ^a^	56.1 ± 2.1 ^b^	36.3 ± 1.3 ^c^
Ethyl isovalerate	7.1 ± 0.4 ^a^	19.1 ± 0.0 ^b^	8.3 ± 0.1 ^a^
Isoamyl acetate	65.6 ± 0.8 ^a^	79.3 ± 1.2 ^b^	52.9 ± 0.8 ^c^
Ethyl hexanoate	114.9 ± 5.1 ^a^	173.7 ± 3.0 ^b^	119.8 ± 14.0 ^a^
Hexyl acetate	2.8 ± 0.2 ^a^	4.3 ± 0.9 ^b^	1.8 ± 0.2 ^c^
Ethyl 2-hydrody-3-methyl butanoate	2.9 ± 0.1 ^a^	10.7 ± 0.6 ^b^	8.9 ± 0.1 ^c^
Ethyl octanoate	215.5 ± 18.4 ^a^	436.9 ± 37.5 ^b^	207.4 ± 6.6 ^a^
Ethyl nonanoate	8.3 ± 0.8 ^a^	15.7 ± 0.2 ^b^	12.6 ± 0.6 ^c^
Ethyl 2-hydroxy-4-methylpentanoate	14.7 ± 2.9 ^a^	26.5 ± 0.1 ^b^	22.0 ± 1.8 ^c^
Isoamyl lactate	14.6 ± 0.6 ^a^	23.6 ± 0.4 ^b^	15.9 ± 0.1 ^a^
Ethyl decanoate	46.6 ± 1.1 ^a^	57.5 ± 2.1 ^b^	48.0 ± 0.2 ^a^
Diethyl succinate	572.6 ± 18.4 ^a^	954.7 ± 57.7 ^b^	460.4 ± 31.8 ^c^
Ethyl 9-decenoate	61.2 ± 1.5 ^a^	80.0 ± 1.6 ^b^	61.4 ± 1.6 ^a^
Ethyl phenylacetate	1.2 ± 0.1 ^a^	3.4 ± 0.2 ^b^	2.3 ± 0.2 ^c^
Phenethyl acetate	54.3 ± 5.0 ^a^	108.8 ± 1.3 ^b^	97.5 ± 3.4 ^c^
Diethyl malate	28.3 ± 0.8 ^a^	47.7 ± 1.6 ^b^	29.6 ± 3.5 ^a^
Ethyl 3-hydroxytridecanoate	28.7 ± 0.1 ^a^	91.5 ± 2.8 ^b^	66.1 ± 4.6 ^c^
Methyl vanillate	1.7 ± 0.1 ^a^	6.2 ± 0.0 ^b^	4.3 ± 0.3 ^c^
Total	1246.6 ± 146.9	13,665.6 ± 133.5	10,039.3 ± 421.2
% esters	0.15%	1.58%	1.35%
***Aldehydes***			
Acetaldehyde	62,513.36 ± 3140.0 ^a^	24,201.6 ± 980.0 ^b^	15,325.5 ± 200.0 ^c^
Benzeneacetaldehyde	77.0 ± 0.7 ^a^	103.3 ± 1.4 ^b^	68.9 ± 1.4 ^c^
Total	62,590.3 ± 3140.7	24,304.9 ± 981.4	15,394.4 ± 201.4
% aldehydes	7.43%	2.80%	2.07%

Different letters in superscript mean significant differences (*p* < 0.05) from two-way ANOVA on the basis of Bonferroni’s multivariate test (BSD).

**Table 2:** Volatile compound concentration (µg/L) in Riesling white wines (control, pollen, and commercial activator).

Volatile Compound	Control	Pollen	Commercial
***Higher alcohols***			
2-Propanol	475,417.7 ± 5480.0 ^a^	489,359.4 ± 23,570.0 ^a^	297,882.7 ± 14,560.0 ^b^
1-Propanol	2090.0 ± 50.0 ^a^	1580.0 ± 60.0 ^b^	2090.0 ± 60.0 ^a^
2-Methyl-1-propanol	13,315.3 ± 223.3 ^a^	11,882.7 ± 580.0 ^b^	13,171.6 ± 236.7 ^a^
3-Methyl-1-butanol	151,227.3 ± 6537.1 ^a^	152,178.0 ± 7425.3 ^a,b^	163,143.3 ± 3056.3 ^b^
Total	642,050.2 ± 12290.4	655,000.2 ± 31,635.3	476,287.6 ± 17,913.0
% higher alcohols	91.64%	89.32%	87.46%
***Methanol***	19,623.5 ± 920.0 ^a^	15,773.9 ± 410.0 ^b^	15,497.5 ± 760.0 ^b^
Total	19,623.5 ± 920.0	15,773.9 ± 410.0	15,497.5 ± 760.0
% methanol	2.80%	2.15%	2.85%
***Acids***			
Butanoic acid	9.6 ± 0.1 ^a^	20.7 ± 0.7 ^b^	26.0 ± 0.7 ^c^
3-Methyl-butanoic acid	94.3 ± 4.3 ^a^	113.1 ± 0.9 ^b^	127.0 ± 3.6 ^b^
Hexanoic acid	1397.3 ± 99.7 ^a^	1407.5 ± 222.8 ^a^	1460.2 ± 181.3 ^a^
Heptanoic acid	22.3 ± 2.3 ^a^	22.7 ± 2.8 ^a^	36.2 ± 6.0 ^b^
2-Hexenoic acid	33.6 ± 0.7 ^a^	55.7 ± 0.3 ^b^	56.8 ± 0.3 ^b^
Octanoic acid	2746.1 ± 153.7 ^a^	1242.1 ± 1.1 ^b^	2928.1 ± 233.2 ^a^
Nonanoic acid	7.2 ± 0.3 ^a^	15.7 ± 0.1 ^b^	19.7 ± 0.8 ^c^
n-Decanoic acid	96.1 ± 2.4 ^a^	197.2 ± 1.5 ^b^	194.1 ± 1.1 ^b^
9-Decenoic acid	89.1 ± 6.7 ^a^	152.9 ± 7.2 ^b^	175.5 ± 4.6 ^c^
Benzoic acid	77.7 ± 2.3 ^a^	122.0 ± 0.3 ^b^	125.8 ± 0.1 ^b^
Phenylacetic acid	8.1 ± 0.2 ^a^	16.0 ± 0.7 ^b^	17.1 ± 0.4 ^b^
Total	4581.4 ± 272.6	3365.5 ± 238.4	5166.5 ± 432.0
% acids	0.65%	0.46%	0.95%
***C-6 Alcohols***			
1-Hexanol	432.1 ± 10.6 ^a^	554.3 ± 10.8 ^b^	459.5 ± 3.6 ^c^
(E)-3-hexen-1-ol	15.0 ± 0.6 ^a^	37.6 ± 0.7 ^b^	29.2 ± 0.7 ^c^
(Z)-3-hexen-1-ol	26.7 ± 2.8 ^a^	106.1 ± 2.5 ^b^	85.0 ± 4.0 ^c^
Total	473.8 ± 14.0	697.9 ± 14.0	573.6 ± 8.4
% C-6 alcohols	0.07%	0.10%	0.11%
***Alcohols***			
3-Penten-2-ol	13.5 ± 1.4 ^a^	23.9 ± 0.7 ^b^	27.9 ± 2.4 ^c^
1-Pentanol	1190.6 ± 81.9 ^a^	1094.6 ± 36.0 ^b^	1245.0 ± 57.6 ^a^
3-Ethyl-2-pentanol	6.3 ± 0.1 ^a^	22.4 ± 0.4 ^b^	24.3 ± 1.7 ^c^
4-Methyl-1-pentanol	13.7 ± 0.8 ^a^	33.3 ± 2.2 ^b^	43.0 ± 1.6 ^c^
3-Methyl-1-pentanol	62.6 ± 4.2 ^a^	156.4 ± 2.0 ^b^	89.3 ± 8.8 ^c^
3-Ethoxy-1-propanol	8.5 ± 0.1 ^a^	9.6 ± 0.2 ^b^	10.7 ± 0.8 ^c^
1-Octanol	36.9 ± 1.4 ^a^	42.5 ± 0.0 ^b^	30.8 ± 1.0 ^c^
1-Nonanol	8.9 ± 0.3 ^a^	27.0 ± 0.3 ^b^	24.6 ± 0.1 ^c^
Benzyl alcohol	54.2 ± 0.6 ^a^	64.8 ± 1.9 ^b^	43.5 ± 1.5 ^c^
2-Phenylethanol	4432.3 ± 189.3 ^a^	1200.7 ± 114.5 ^b^	1047.5 ± 14.1 ^b^
1H-Indole-3-ethanol	617.2 ± 0.3 ^a^	116.6 ± 2.4 ^b^	104.2 ± 1.4 ^b^
Total	6444.6 ± 280.3	2791.7 ± 160.5	2690.6 ± 91.0
% alcohols	0.92%	0.38%	0.49%
***Terpenes***			
Linalool oxide	9.0 ± 0.2 ^a^	16.3 ± 0.8 ^b^	11.7 ± 0.7 ^c^
Linalool	4.5 ± 0.3 ^a^	15.1 ± 1.5 ^b^	11.1 ± 0.2 ^c^
α-Terpineol	12.2 ± 0.7 ^a^	26.8 ± 0.1 ^b^	17.2 ± 0.7 ^c^
β-Citronellol	4.5 ± 0.2 ^a^	16.5 ± 1.1 ^b^	16.0 ± 0.3 ^b^
2,6-Dimethyl-3,7-octadiene-2,6-diol	70.4 ± 0.1 ^a^	94.6 ± 1.7 ^b^	66.3 ± 4.5 ^a^
8-Hydroxilinalool	6.7 ± 0.4 ^a^	24.2 ± 1.6 ^b^	22.5 ± 2.0 ^b^
Total	107.4 ± 1.9	193.4 ± 6.8	144.8 ± 8.4
% terpenes	0.02%	0.03%	0.03%
***Esters***			
Ethyl acetate	17,797.2 ± 590.0 ^a^	12,172.1 ± 314.1^b^	13,628.0 ± 80.0 ^c^
Ethyl butyrate	7.3 ± 0.1 ^a^	25.8 ± 0.2 ^b^	21.3 ± 1.2 ^c^
Ethyl isovalerate	2.1 ± 0.1 ^a^	10.9 ± 0.5 ^b^	9.7 ± 0.4 ^c^
Isoamyl acetate	111.2 ± 11.4 ^a^	589.9 ± 4.5 ^b^	560.6 ± 53.0 ^b^
Ethyl hexanoate	51.0 ± 0.6 ^a^	244.2 ± 3.1 ^b^	159.5 ± 11.5 ^c^
Hexyl acetate	2.7 ± 0.2 ^a^	13.6 ± 0.8 ^b^	13.2 ± 0.3 ^b^
Ethyl 2-hydrody-3-methyl butanoate	4.2 ± 0.4 ^a^	16.1 ± 0.4 ^b^	12.6 ± 1.0 ^c^
Ethyl octanoate	565.6 ± 13.4 ^a^	757.3 ± 55.7 ^b^	410.1 ± 5.4 ^c^
Ethyl nonanoate	9.0 ± 0.2 ^a^	17.0 ± 0.6 ^b^	14.0 ± 0.2 ^c^
Ethyl 2-hydroxy-4-methylpentanoate	19.8 ± 1.6 ^a^	60.0 ± 4.4 ^b^	58.9 ± 5.7 ^b^
Isoamyl lactate	21.2 ± 0.7 ^a^	43.0 ± 1.2 ^b^	21.7 ± 2.1 ^a^
Ethyl decanoate	55.0 ± 2.6 ^a^	124.9 ± 10.9 ^b^	121.6 ± 3.5 ^b^
Diethyl succinate	663.0 ± 0.1 ^a^	1435.3 ± 63.4 ^b^	871.8 ± 83.3 ^c^
Ethyl 9-decenoate	13.1 ± 0.3 ^a^	32.3 ± 0.1 ^b^	29.3 ± 0.8 ^c^
Ethyl phenylacetate	2.0 ± 0.2 ^a^	4.1 ± 0.1 ^b^	2.4 ± 0.2 ^c^
Phenethyl acetate	58.7 ± 1.0 ^a^	242.7 ± 14.4 ^b^	170.6 ± 8.7 ^c^
Diethyl malate	30.8 ± 0.7 ^a^	60.7 ± 0.6 ^b^	40.4 ± 0.8 ^c^
Ethyl 3-hydroxytridecanoate	10.5 ± 0.1 ^a^	24.1 ± 1.2 ^b^	16.7 ± 1.4 ^c^
Methyl vanillate	18.6 ± 0.3 ^a^	46.8 ± 0.6 ^b^	36.3 ± 4.2 ^c^
Total	1645.8 ± 624.0	15,920.5 ± 476.8	16,198.7 ± 263.5
% esters	0.23%	2.17%	2.97%
***Aldehydes***			
Acetaldehyde	25,673.4 ± 13,60.0 ^a^	39,435.7 ± 1410.0 ^b^	27,900.3 ± 1280.0 ^a^
Benzeneacetaldehyde	30.0 ± 0.1 ^a^	101.8 ± 1.0 ^b^	97.8 ± 1.6 ^b^
Total	25,703.4 ± 1360.1	39,537.5 ± 1411.0	27,998.1 ± 1281.6
% aldehydes	3.67%	5.39%	5.14%

Different letters in superscript mean significant differences (*p* < 0.05) from two-way ANOVA on the basis of Bonferroni’s multivariate test (BSD).

**Table 3:** Volatile compound concentration (µg/L) in Tintilla de Rota red wines (control, pollen, and commercial activator).

Volatile compound	Control	Pollen	Commercial
***Higher alcohols***			
2-Propanol	293,773.7 ± 13,302.9 ^a^	379,849.2 ± 14,140.0 ^b^	302,480.2 ± 8707.4 ^a^
1-Propanol	1306.8 ± 20.0 ^a^	4529.0 ± 20.0 ^b^	3141.0 ± 60.0 ^c^
2-Methyl-1-propanol	23,210.1 ± 500.0 ^a^	19,253.6 ± 760.0 ^b^	20,531.5 ± 820.0 ^b^
3-Methyl-1-butanol	246,833.3 ± 3329.3 ^a^	276,725.2 ± 6681.7 ^b^	273,487.4 ± 10,275.4 ^b^
Total	565,123.9 ± 17,152.3	680,357.1 ± 21601.7	599,640.2 ± 19,862.8
% higher alcohols	80.20%	86.04%	84.34%
***Methanol***	63,489.4 ± 3570.0 ^a^	55,928.6 ± 1160.0 ^b^	61,709.1 ± 1139.8 ^a^
Total	63,489.4 ± 3570.0	55,928.6 ± 1160.0	61,709.1 ± 1139.8
% methanol	9.01%	7.07%	8.68%
***Acids***			
Butanoic acid	29.4 ± 0.7 ^a^	30.7 ± 0.7 ^a^	33.8 ± 0.4 ^b^
3-Methyl-butanoic acid	190.4 ± 2.6 ^a^	177.2 ± 2.4 ^a^	181.2 ± 1.4 ^a^
Hexanoic acid	572.4 ± 24.4 ^a^	446.0 ± 59.8 ^b^	370.9 ± 18.2 ^c^
Heptanoic acid	26.0 ± 0.9 ^a^	23.3 ± 0.7 ^b^	27.2 ± 0.9 ^a^
2-Hexenoic acid	34.1 ± 1.2 ^a^	38.4 ± 0.7 ^b^	40.1 ± 0.1 ^b^
Octanoic acid	1476.8 ± 93.5 ^a^	883.8 ± 68.8 ^b^	1185.9 ± 19.7 ^c^
Nonanoic acid	88.5 ± 0.1 ^a^	87.4 ± 2.5 ^a^	88.3 ± 2.5 ^a^
n-Decanoic acid	726.9 ± 7.4 ^a^	362.2 ± 32.0 ^b^	403.7 ± 15.4 ^b^
9-Decenoic acid	36.2 ± 1.9 ^a^	41.4 ± 0.7 ^b^	43.3 ± 2.7 ^b^
Benzoic acid	71.6 ± 1.4 ^a^	130.5 ± 14.1 ^b^	134.6 ± 15.2 ^b^
Phenylacetic acid	46.1 ± 3.6 ^a^	39.6 ± 2.3 ^b^	52.8 ± 0.6 ^c^
Total	3298.4 ± 137.6	2260.6 ± 184.7	2561.8 ± 77.0
% acids	0.47%	0.29%	0.36%
***C-6 Alcohols***			
1-Hexanol	405.8 ± 3.1 ^a^	367.5 ± 34.7 ^a^	325.6 ± 16.5 ^b^
(E)-3-Hexen-1-ol	15.9 ± 0.4 ^a^	31.1 ± 1.6 ^b^	27.0 ± 0.7 ^c^
(Z)-3-Hexen-1-ol	23.9 ± 0.8 ^a^	26.7 ± 1.2 ^b^	22.4 ± 1.4 ^a^
Total	445.5 ± 4.3	425.3 ± 37.5	375.0 ± 18.5
% C-6-alcohols	0.06%	0.05%	0.05%
***Alcohols***			
3-Penten-2-ol	14.1 ± 1.1 ^a^	27.9 ± 0.4 ^b^	32.3 ± 1.8 ^c^
1-Pentanol	1492.9 ± 119.4 ^a^	1338.7 ± 28.2 ^b^	1378.0 ± 35.7 ^a,b^
3-Ethyl-2-pentanol	11.9 ± 2.1 ^a^	13.1 ± 1.0 ^a^	18.4 ± 1.4 ^b^
4-Methyl-1-pentanol	15.6 ± 0.5 ^a^	15.8 ± 0.2 ^a^	21.5 ± 0.5 ^b^
3-Methyl-1-pentanol	266.1 ± 23.0 ^a^	130.3 ± 2.4 ^b^	83.6 ± 2.9 ^c^
3-Ethoxy-1-propanol	87.0 ± 0.9 ^a^	123.3 ± 2.5 ^b^	111.4 ± 1.9 ^c^
1-Octanol	24.4 ± 1.7 ^a^	25.1 ± 0.5 ^a^	28.8 ± 0.9 ^b^
1-Nonanol	3.7 ± 0.2 ^a^	2.2 ± 0.2 ^b^	1.9 ± 0.1 ^b^
Benzyl alcohol	80.7 ± 0.8 ^a^	140.4 ± 7.5 ^b^	103.5 ± 11.6 ^c^
2-Phenylethanol	2440.2 ± 6.0 ^a^	2597.6 ± 133.5 ^a^	2128.8 ± 6.6 ^b^
1H-Indole-3-ethanol	854.8 ± 4.2 ^a^	662.0 ± 40.6 ^b^	673.8 ± 50.8 ^b^
1-Butanol	32.7 ± 3.0 ^a^	18.5 ± 0.9 ^b^	10.4 ± 0.4 ^c^
3-Methyl-2-buten-1-ol	47.0 ± 1.1 ^a^	34.3 ± 2.2 ^b^	32.7 ± 1.7 ^b^
Total	5371.2 ± 164.1	5129.2 ± 220.2	4625.2 ± 116.2
% alcohols	0.76%	0.65%	0.65%
***Phenols***			
2,6-Di-tert-butyl-4-ethylphenol	17.9 ± 0.1 ^a^	30.6 ± 1.2 ^b^	27.3 ± 3.3 ^c^
4-Ethylphenol	5.8 ± 0.1 ^a^	6.4 ± 0.1 ^a^	5.8 ± 0.1 ^a^
4-Vinylguaiacol	36.8 ± 1.7 ^a^	64.2 ± 1.5 ^b^	53.1 ± 0.6 ^c^
Acetovanillone	89.3 ± 2.1 ^a^	87.9 ± 5.5 ^a^	61.2 ± 6.9 ^b^
Total	149.9 ± 4.0	189.1 ± 8.3	147.4 ± 10.8
% phenols	0.02%	0.02%	0.02%
***Terpenes and derivatives***			
Linalool oxide	23.3 ± 1.6 ^a^	65.4 ± 0.5 ^b^	60.3 ± 4.4 ^b^
Linalool	7.9 ± 0.6 ^a^	14.3 ± 0.1 ^b^	15.1 ± 1.3 ^b^
α-Terpieol	8.9 ± 0.4 ^a^	15.3 ± 0.9 ^b^	12.8 ± 0.2 ^c^
β-Citronellol	8.2 ± 0.1 ^a^	10.8 ± 0.1 ^b^	10.3 ± 0.7 ^b^
2,6-Dimetil-3,7-octadiene-2,6-diol	30.5 ± 2.0 ^a^	45.6 ± 1.5 ^b^	39.9 ± 3.5 ^c^
8-Hydroxylinalool	50.4 ± 3.5 ^a^	194.1 ± 5.6 ^b^	92.2 ± 3.0 ^c^
Total	129.1 ± 8.2	345.6 ± 8.7	230.6 ± 13.0
Terpenes and derivatives	0.02%	0.04%	0.03%
***Esters***			
Ethyl acetate	34,390.6 ± 1370.0 ^a^	25,802.0 ± 590.0 ^b^	24,869.0 ± 530.0 ^b^
Ethyl butyrate	24.5 ± 1.3 ^a^	72.8 ± 1.3 ^b^	54.5 ± 4.0 ^c^
Ethyl isovalerate	27.4 ± 0.5 ^a^	30.2 ± 1.9 ^b^	15.7 ± 0.4 ^c^
Isoamyl acetate	67.1 ± 3.3 ^a^	76.2 ± 1.1 ^b^	30.7 ± 1.0 ^c^
Ethyl hexanoate	79.5 ± 2.4 ^a^	157.9 ± 12.9 ^b^	115.6 ± 4.2 ^c^
Hexyl acetate	35.4 ± 1.8 ^a^	76.6 ± 0.9 ^b^	62.7 ± 1.0 ^c^
Butanoic acid, 2-hydroxy-3-methyl-, ethyl ester	5.7 ± 0.3 ^a^	16.8 ± 0.3 ^b^	13.9 ± 0.8 ^c^
Ethyl octanoate	333.1 ± 12.2 ^a^	415.0 ± 32.5 ^b^	317.2 ± 22.0 ^a^
Ethyl nonanoate	10.1 ± 0.1 ^a^	11.5 ± 0.4 ^b^	10.4 ± 0.1 ^a^
Ethyl 2-hydroxy-4-methylpentanoate	32.4 ± 2.3 ^a^	43.0 ± 3.5 ^b^	49.3 ± 0.1 ^c^
Isoamyl lactate	48.0 ± 2.1 ^a^	239.9 ± 4.4 ^b^	208.0 ± 4.7 ^c^
Ethyl decanoate	214.7 ± 5.6 ^a^	278.6 ± 6.3 ^b^	273.9 ± 16.3 ^b^
Diethyl succinate	404.0 ± 17.0 ^a^	971.2 ± 40.7 ^b^	944.7 ± 66.2 ^b^
Ethyl 9-decenoate	58.7 ± 0.6 ^a^	63.9 ± 3.8 ^b^	58.8 ± 0.2 ^a^
Ethyl phenylacetate	1.2 ± 0.0 ^a^	2.2 ± 0.0 ^b^	2.4 ± 0.2 ^c^
Phenethyl acetate	35.8 ± 1.4 ^a^	140.4 ± 8.3 ^b^	138.0 ± 5.7 ^b^
Diethyl malate	22.4 ± 0.7 ^a^	34.7 ± 1.6 ^b^	30.0 ± 0.3 ^c^
Methyl vanillate	147.7 ± 12.2 ^a^	583.1 ± 8.8 ^b^	404.3 ± 0.4 ^c^
Ethyl lactate	121.6 ± 1.6 ^a^	153.5 ± 1.3 ^b^	151.0 ± 3.7 ^b^
Butanoic acid 3-hydroxy, ethyl ester	56.1 ± 0.3 ^a^	89.3 ± 6.3 ^b^	73.3 ± 4.8 ^c^
Ethyl (Z)-4-decenoate	56.8 ± 2.1 ^a^	220.3 ± 2.4 ^b^	128.3 ± 7.4 ^c^
Ethyl dodecanoate	57.1 ± 1.0 ^a^	65.2 ± 0.1 ^b^	61.3 ± 4.3 ^a,b^
Methyl tetradecanoate	36.2 ± 0.8 ^a^	84.4 ± 6.4 ^b^	66.8 ± 1.6 ^c^
Succinic acid, 2-hydroxy-3-methyl-, diethyl ester	88.7 ± 1.6 ^a^	130.9 ± 1.9 ^b^	122.2 ± 2.9 ^b^
Methyl hexadecanoate	77.2 ± 2.0 ^a^	154.3 ± 1.0 ^b^	122.8 ± 6.8 ^c^
Hexadecanoic acid, ethyl ester	370.1 ± 25.1 ^a^	754.2 ± 5.8 ^b^	354.9 ± 10.5 ^a^
Propanoic acid, 2-methyl, propyl, 2-methyl, ester	79.7 ± 0.6 ^a^	166.0 ± 0.5 ^b^	143.3 ± 4.8 ^c^
Ethyl 8-nonenoate	201.6 ± 0.7 ^a^	245.4 ± 2.9 ^b^	220.4 ± 1.1 ^c^
Total	2692.6 ± 1469.7	31,079.5 ± 747.4	29,043.3 ± 705.2
% esters	0.38%	3.93%	4.08%
***Aldehydes***			
Acetaldehyde	63,723.2 ± 2330.0 ^a^	14,546.3 ± 458.5 ^b^	12,209.5 ± 520.0 ^b^
Benzeneacetaldehyde	63.6 ± 4.7 ^a^	123.8 ± 2.2 ^b^	120.6 ± 0.9 ^b^
Nonanal	10.0 ± 0.2 ^a^	16.5 ± 1.2 ^b^	12.8 ± 0.6 ^c^
3-Methyl-butanal	13.6 ± 1.6 ^a^	30.1 ± 0.2 ^b^	26.3 ± 1.1 ^c^
Total	63,810.4 ± 2336.5	14,716.6 ± 462.1	12,369.2 ± 522.7
% aldehydes	9.06%	1.86%	1.74%
***Thiols***			
3-(Methylthio)-1-propanol	18.1 ± 1.6 ^a^	121.7 ± 1.9 ^b^	104.3 ± 3.3 ^c^
Total	18.1 ± 1.6	121.7 ± 1.9	104.3 ± 3.3
% thiols	0.003%	0.02%	0.01%
***Acetals***			
1-(1-(1-Ethoxyethoxy)-pentane	1.9 ± 0.1 ^a^	2.0 ± 0.1 ^a^	2.1 ± 0.2 ^a^
Total	1.9 ± 0.1	2.0 ± 0.1	2.1 ± 0.2
% acetals	0.0003%	0.0003%	0.0003%
***Norisoprenoids***			
3-Oxo-α-ionol	5.9 ± 0.1 ^a^	12.2 ± 1.1 ^b^	15.1 ± 1.0 ^c^
Total	5.9 ± 0.1	12.2 ± 1.1	15.1 ± 1.0
% norisoprenoids	0.0008%	0.002%	0.002%
***Lactones***			
Dihydro-5-pentyl-2(3H)-furanone	56.1 ± 2.4 ^a^	108.4 ± 0.4 ^b^	99.7 ± 1.6 ^c^
2,3-Dihydro-benzofuran	45.5 ± 1.8 ^a^	45.8 ± 1.0 ^a^	55.1 ± 0.2 ^b^
Total	101.6 ± 4.2	154.2 ± 1.4	154.8 ± 1.8
% lactones	0.0144%	0.0219%	0.0220%

Different letters in superscript mean significant differences (*p* < 0.05) from two-way ANOVA on the basis of Bonferroni’s multivariate test (BSD).

**Table 4:** Main component loads of volatile organic compounds in white and red wines (control, commercial activator, and bee pollen).

Volatile Compounds	F1	F2	F3
Higher alcohols	−0.097	0.009	0.986
Methanol	0.875	−0.155	0.195
Acids	−0.669	−0.535	−0.081
C-6 alcohols	−0.754	−0.144	0.476
Alcohols	0.356	−0.704	0.387
Terpenes and derivatives	0.668	0.614	0.068
Esters	0.497	0.834	−0.024
Aldehydes	−0.086	−0.832	−0.052
Thiols	0.873	0.418	0.059
Acetals	0.980	0.061	−0.148
Norisoprenoids	0.938	0.274	−0.062
Lactones	0.978	0.178	−0.089
Phenols	0.977	0.083	−0.108
Explained variance (%)	54.88	22.19	11.16

**Table 5:** Sum of the odorant activity values (ΣOAV) of Palomino Fino and Riesling white wines, grouped by odorant series.

	Palomino Fino			Riesling		
Odorant Series	Control	Pollen	Commercial	Control	Pollen	Commercial
Fruity	85.74	154.02	85.69	138.28	239.97	149.42
Floral	17.63	24.61	16.91	8.56	25.42	23.23
Fatty	17.09	11.28	14.01	11.83	9.59	13.57
Grassy	1.06	1.03	1.05	0.58	0.99	0.81
Dried fruit	6.24	2.43	1.54	2.56	3.93	2.78
Earthy, mushroom	0.30	0.28	0.29	0.15	0.31	0.19
Chemical	0.09	0.09	0.10	0.08	0.12	0.13
Spicy	0.03	0.05	0.04	0.01	0.02	0.05
ΣOAV_T_	128.18	193.79	119.62	162.05	280.36	190.17

**Table 6:** Sum of odorant activity values (ΣOAV) of Tintilla de Rota red wines, grouped by odorant series.

Odorant Series	Control	Pollen	Commercial
Fruity	125.11	173.85	133.14
Floral	17.61	37.74	36.23
Fatty	11.00	8.76	9.36
Grassy	0.54	0.64	0.54
Dried fruit	6.38	1.45	1.22
Earthy, mushroom	0.27	0.26	0.19
Chemical	0.08	0.13	0.13
Spicy	1.06	1.88	1.53
Phenolic	0.01	0.01	0.01
ΣOAV_T_	162.05	224.71	182.35

**Table 7:** Results of the classification test on white and red wines from the comparative study with pollen and commercial activator.

Variety	Control	Pollen	Commercial	Testers	F-Test	Significance (α)
Palomino Fino	42 (24.1%)	66 (37.9%)	66 (37.9%)	29	13.24	0.01
Riesling	49 (28.2%)	72 (41.4%)	53 (30.5%)	29	10.41	0.01
Tintilla de Rota	41 (23.6%)	75 (43.1%)	58 (33.3%)	29	19.93	0.01

(% score in relation to the total).

**Table 8:** Triangular test alpha risk (α) results obtained in the comparative study with pollen and commercial activator.

Variety	Control vs. Pollen	Control vs. Commercial	Pollen vs. Commercial	Testers
Palomino Fino	n.d. (32.1%)	0.2 (42.9%)	0.01 (57.1%)	28
Riesling	0.2 (44.4%)	0.01 (66.7%)	0.2 (44.4%)	27
Tintilla de Rota	0.1 (50.0%)	0.2 (46.2%)	0.2 (46.2%)	26

n.d. (non-detected) (% tasters capable of recognizing the different wines).

## Data Availability

Not applicable.
